# Exacerbation of acidosis during ischemia and reperfusion arrhythmia in hearts from type 2 Diabetic Otsuka Long-Evans Tokushima Fatty rats

**DOI:** 10.1186/1475-2840-6-17

**Published:** 2007-06-05

**Authors:** Ryuko Anzawa, Shingo Seki, Kazuaki Horikoshi, Masayuki Taniguchi, Seibu Mochizuki

**Affiliations:** 1 Division of Cardiology, Department of Internal Medicine, The Jikei University School of Medicine, Tokyo, Japan

## Abstract

**Background:**

Sensitivity to ischemia and its underlying mechanisms in type 2 diabetic hearts are still largely unknown. Especially, correlation between reperfusion induced ventricular arrhythmia and changes in intracellular pH has not been elucidated.

**Methods and results:**

Male Otsuka Long-Evans Tokushima Fatty (OLETF) rats at 16 and 32 weeks of age were used along with age-matched nondiabetic Long-Evans Tokushima Otsuka (LETO) rats. Hearts from rats in these 4 groups were perfused in the working heart mode, thus inducing whole heart ischemia. At 16 weeks of age, no differences in blood glucose levels or incidence and duration of reperfusion arrhythmia were found between the strains. At 32 weeks of age, both impaired glucose tolerance and obesity were observed in the OLETF rats. Further, the duration of reperfusion-induced ventricular fibrillation (VF) was significantly longer in the OLETF rats, while the pH level was significantly lower and proton contents were significantly higher in coronary effluent during ischemia in those rats. Following treatment with troglitazone, improvements in pH and proton level in coronary effluent during ischemia were observed, as was the duration of reperfusion-induced VF in OLETF rats at 32 weeks of age.

**Conclusion:**

The hearts of spontaneously diabetic OLETF rats were found to be more susceptible to ischemic insult. Troglitazone treatment improved ischemic tolerance by improving glucose metabolism in the myocardium of those rats.

## Background

Diabetes mellitus (DM) accelerates changes in athelosclerosis, which leads to an increase in both clinical incidence of ischemic heart disease and mortality rate [1]. Further, independent of coronary artery disease, type 2 DM has direct adverse effects on the myocardium [2]. To elucidate the mechanisms of these actions, recently, echocardiographical study [3] or some whole heart perfusion experiments using type 2 diabetic model rodents have been performed. Alterations in energy metabolism in the myocardium of type 2 diabetic rodents have been well investigated [4-6], whereas ion homeostasis has been rarely investigated [7] in ischemia-reperfusion study.

Otsuka Long-Evans Tokushima Fatty (OLETF) strain is a spontaneously type 2 DM model rat with diabetic complications [8] that shows late onset hyperglycemia at 18 weeks of age. In the present study, we induced ischemia in perfused OLETF rat hearts in order to examine the changes in pH and proton production, during and after ischemia, as well as the incidence and duration of ventricular arrhythmia after reperfusion. Furthermore, we observed if improvement in diabetic state by troglitazone [9], a thiazolidinedione, acts to the ischemic injury in heart from diabetic OLETF rat.

## Methods

### Experimental groups

The present study was undertaken in accordance with the Animals (Scientific Procedures) Act 1986 and conforms with the Guide for the Care and Use of Laboratory Animals published by US National Institutes of Health (NIH Publication No. 85-23, revised 1996).

Male OLETF rats at 6 weeks of age, age-matched non-diabetic Long-Evans Tokushima Otsuka (LETO) rats [8] were obtained from the Otsuka Pharmacology Laboratory (Tokushima, Japan). All of these rats were maintained at the Jikei University animal experiment center and were kept under controlled temperatures (21 ± 2°C) with a 12-hour artificial light and dark cycle. These rats were then divided into 6 subgroups: OLETF at 16 weeks of age (16-O, n = 8), LETO at 16 weeks of age (16-L, n = 8), OLETF at 32 weeks of age (32-O, n = 8), LETO at 32 weeks of age (32-L, n = 8), OLETF at 32 weeks of age treated with troglitazone (32-OT, n = 8), and LETO at 32 weeks of age treated with troglitazone (32-LT, n = 7). Rat standard laboratory chow (type MF, Oriental Yeast Co) with or without troglitazone (0.2% vol/vol) was given ad libitium from ages 24 to 32 weeks.

### Body weights

The body weight (BW) was measured immediately before each heart perfusion experiment.

### Oral glucose tolerance test

The oral glucose tolerance test (OGTT) was performed in all groups within a week before each perfusion experiment. After 18 hours of fasting, glucose fluid (2 g/kg BW) was administered using a gastric tube. Blood samples were obtained via the tail vein at 3 time points: before the administration of glucose fluid (pre), 60 minutes after loading, and120 minutes after loading.

### Heart perfusion and ischemia

Rats in all groups were anesthetized with sodium pentobarbital (50 mg/kg, i.p. injection) and a thoracotomy was performed after 18 hours of fasting. Blood samples were obtained from the inferior vena cava for measurements of glycohemoglobin A1c (HbA1c) levels and serum levels of insulin. The hearts were quickly removed and then immersed in ice-cold Krebs-Henseleit bicarbonate solution. The aorta and the pulmonary vein were cannulated, and Langendorff's retrograde perfusion was initiated at 37°C and a hydrostatic pressure of 80 mmHg. After 10 min, Langendorff perfusion was switched to the physiological perfusion, working heart mode by clamping the aortic inflow line from the Langendorff reservoir, and opening the left atrial inflow and aortic line. The afterload was maintained at 60 mmHg of hydrostatic pressure and the preload was maintained at 7 mmHg throughout the experiment. Modified Krebs-Henseleit bicarbonate buffer containing (in mmol) NaCl 118, KCl 4.7, NaHCO_3 _2.5, CaCl_2 _2.5, MgSO_4_·H_2_O 1.2, EDTA 0.5, KH_2_PO_4 _1.2 and glucose 11 (37°C, pH 7.4) bubbled with 95% O_2_and 5% CO_2 _was used as the perfusion buffer. Subsequent to the 5 min control perfusion, whole heart ischemia (flow rate 5–10%) was induced by the use of a one-way ball valve which prevented retrograde perfusion during diastole and the hearts were then perfused with electrical pacing (300 beats/min) for 10 min. After reperfusion, the pacing and the ball valve action were stopped for an additional 20 min.

### Hemodynamic measurements and analysis of the coronary effluent

The aortic flow was measured with an electromagnetic flowmeter (Nihon-Koden MFV 2100, Tokyo). An 18-gauge catheter was inserted via the left atrium into the left ventricle to measure the left ventricular (LV) pressure, the peak positive first derivative of LV pressure (LV +dP/dt) and peak negative first derivative of LV pressure (LV -dP/dt) with a polygraph system (Fukuda Denshi Co., Tokyo, MIC 8600). An electrocardiogram (ECG) recorded from an epicardium was monitored using carbon lead attached to the surface of the heart. The coronary flow was collected by a heart chamber at 5 min intervals throughout the period of perfusion. Cardiac output was estimated as the sum of the aortic and coronary flow.

The coronary effluent was collected from the pulmonary artery and used for determination of venous PO_2_, PCO_2_, HCO_3 _^- ^and pH levels with a blood gas analyzer (Corning 175, USA).

### Arrhythmia study

ECG was continuously recorded on a recorder throughout the experiment. The ECG data were retrospectively analyzed, in a blind manner, for the incidence, time to onset, and duration of ventricular fibrillation (VF) during reperfusion. All analyses were carried out in accordance with the Lambeth Conventions [10]. VF was defined as a signal in which individual QRS deflections could no longer be distinguished from one another and the heart rate could not be determined.

### Biochemical analysis

The blood glucose level was measured in the whole blood using glucose oxidase method. HbA1c was measured by the latex method. The plasma insulin level was measured using the enzyme-immunoassay insulin kits (Morinaga, Tokyo Japan).

The proton production in coronary effluent was calculated from the PCO_2 _and HCO_3 _^- ^values as follows: [H^+^] (n mol/l) = 24 × PCO_2 _(mmHg)/[HCO_3 _^-^] (m mol/l)

### Stastical analysis

Values are expressed as the mean ± standard error. The data were analyzed using either Student's t-test for unpaired data or analysis of variance followed by appropriate post-hoc test to locate differences between groups. Binominal distributed variables, such as the incidence of VF, were compared using the χ^2 ^test for a 2 × n table, followed by a sequence of 2 × 2χ^2 ^tests with Yates's correction. A value of p < 0.05 was considered to be statistically significant.

## Results

### Characteristics of OLETF and LETO rats

Body weight was significantly higher in 16-O compared with that in 16-L, while 32-O seemed to be obese, with body weight significantly higher as compared with 32-L and 16-O. No significant differences were seen in body weights among the groups based on troglitazone treatment. The level of HbA1c showed no difference between 16-L and 16-O, however, that in 32-O was significantly higher than that in 32-L. HbA1c in 32-OT was significantly lower as compared with 32-O, while there was no significant difference between 32-LT and 32-L. The plasma glucose level in 16-O was higher than that in 16-L before OGTT, however, there were no differences between those 2 groups in regard to OGTT results. The level of plasma glucose in 32-O showed a significantly higher level than that in 32-L before and during OGTT, while a significant improvement in plasma glucose level in 32-OT was observed following treatment with troglitazone before and in OGTT results. In contrast, troglitazone did not have an affect on plasma glucose level in 32-LT. The serum insulin level showed no significant difference between 16-O and 16-L, however, that in 32-O tended to be higher than that in 32-L (p= 0.08). Hyperinsulinemia seen in 32-O improved slightly, as shown in 32-OT (p = 0.10), by treatment with troglitazone. These results are shown in Table 1.

**Table 1:** Characteristics of the LETO and OLETF rats in each groups

	16-L	16-O	32-L	32-O	32-LT	32-OT
	(n = 8)	(n = 8)	(n = 8)	(n = 8)	(n = 7)	(n = 8)
Body Weight (g)	383.8 ± 8.9	468.8 ± 10.6 **	460.8 ± 7.6	563.2 ± 15.2^††^	465.0 ± 14.9	521.4 ± 21.5
HbA1c (%)	3.04 ± 0.07	3.18 ± 0.06	3.17 ± 0.07	4.45 ± 0.26^††^	3.09 ± 0.13	3.52 ± 0.07^#^
Serum Insulin (ng/ml)	1.5 ± 2.3	2.4 ± 0.8	1.7 ± 0.9	3.3 ± 1.1	1.6 ± 0.6	1.8 ± 0.2
Plasma glucose in OGTT (mg/dl)						
pre	86.6 ± 2.2	100.9 ± 4.1*	106.3 ± 3.7	122.3 ± 5.1^†^	95.8 ± 2.0	98.8 ± 3.9^#^
60 min	131.1 ± 4.1	157.1 ± 14.4	142.5 ± 4.1	310.7 ± 5.0^††^	134.9 ± 1.7	225.6 ± 6.9^##^
120 min	102.6 ± 4.1	100.9 ± 1.9	101.9 ± 1.9	161.5 ± 4.8^††^	95.1 ± 3.1	98.9 ± 4.3^##^

Values are the means ± SEM. HbA1c, glycohemoglobin A1c; OGTT, oral glucose tolerance test.

*p < 0.05 vs 16-L, **p < 0.01 vs 16-L, ^†^p < 0.05 vs 32-L, ^††^p < 0.01 vs 32-L, ^#^p < 0.05 vs 32-O, ^## ^p < 0.01 vs 32-O

### Basal cardiac function

In the perfused working hearts, there were no differences in the heart rate, coronary flow, cardiac output, LV pressure, and LV ± dP/dt between 16-O and 16-L. The effects of aging in these parameters were not seen between 32-O and 16-O, and between 32-L and 16-L. In addition, there were no significant differences in these parameters between 32-OT and 32-O, or between 32-LT and 32-L. These results are shown in Table 2.

**Table 2:** Myocardial function before ischemia in each group.

	16-L (n = 8)	16-O (n = 8)	32-L (n = 8)	32-O (n = 8)	32-LT (n = 7)	32-OT (n = 6)
HR (beats/min)	212.3 ± 13.2	202.5 ± 7.7	219.1 ± 7.7	202.8 ± 10.7	218.2 ± 5.6	201.0 ± 9.3
CF (ml/min)	16.0 ± 1.0	17.1 ± 1.0	19.5 ± 0.5	21.1 ± 0.8	21.2 ± 0.7	21.7 ± 0.4
CO (ml/min)	45.1 ± 3.5	52.9 ± 2.2	51.1 ± 3.0	50.4 ± 4.0	58.1 ± 2.7	51.0 ± 2.8
LVP (mmHg)	133.0 ± 3.0	142.8 ± 4.6	135.5 ± 4.9	134.1 ± 6.3	140.1 ± 1.9	130.7 ± 2.8
LV +dP/dt (mmHg/sec × 10^3^)	2.6 ± 0.3	2.9 ± 0.2	4.0 ± 0.2	3.6 ± 0.2	4.1 ± 0.1	3.4 ± 0.1
LV -dP/dt (mmHg/sec × 10^3^)	2.5 ± 0.2	2.5 ± 0.3	3.2 ± 0.3	2.8 ± 0.3	3.3 ± 0.3	3.0 ± 0.2

Values are mean SEM. HR, Heart rate; CF, Coronary flow; CO, Cardiac output; LVP, Left ventricular pressure; LV +dP/dt, Peak positive first derivative of left ventricular pressure; LV -dP/dt, Peak negative first derivative of left ventricular pressure.

### Reperfusion induced arrhythmia

**Figure 1:**
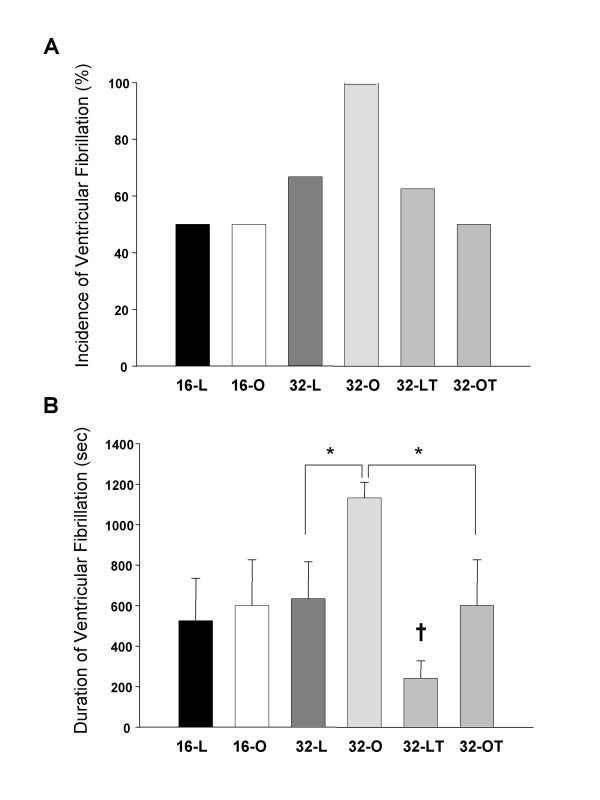
Incidence (A) and duration (B) of ventricular fibrillation after reperfusion. * p < 0.05 vs. OLETF rats at 32 weeks of age, † p < 0.05 vs. LETO rats at 32 weeks of age.

Reperfusion induced arrhythmia VF occurred during the early phase of reperfusin after ischemia. There were no significant differences in the incidence of VF between OLETF and age-matched LETO rats. The incidence of VF (Fig. 1A) was 50% in both 16-O and 16-L, while that in 32-O was 100%, though it decreased (32-OT: 50%, p = 0.10) by treatment with troglitazone. There was no significant difference in the duration of VF (Fig. 1B) between 16-O and 16-L. That in 32-O was significantly longer than in 32-L. The duration of VF in 32-O was significantly shortened by treatment with troglitazone, and that in 32-L was also significantly shortened as compared with 32-LT.

### Changes in pH of coronary effluent

**Figure 2:**
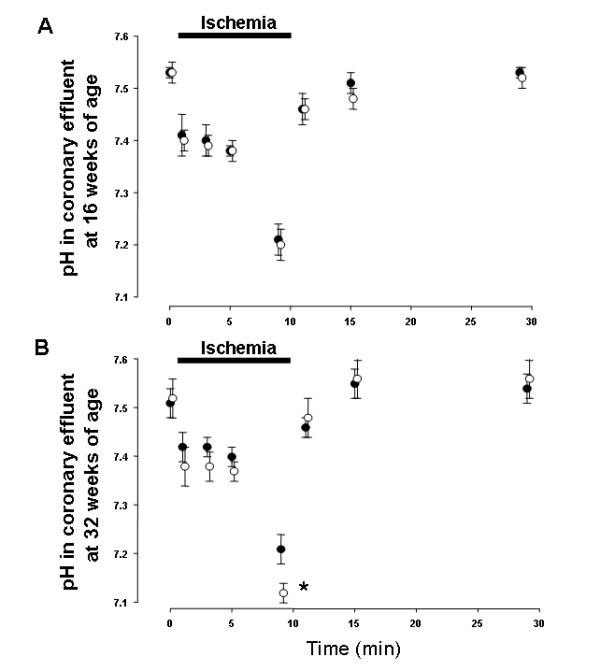
Changes in pH of coronary effluent in LETO and OLETF rats at 16 weeks of age (A) and 32 weeks of age (B). Closed circles show LETO rats, open circles show OLETF rats. * p < 0.05 vs. LETO rats.

**Figure 3:**
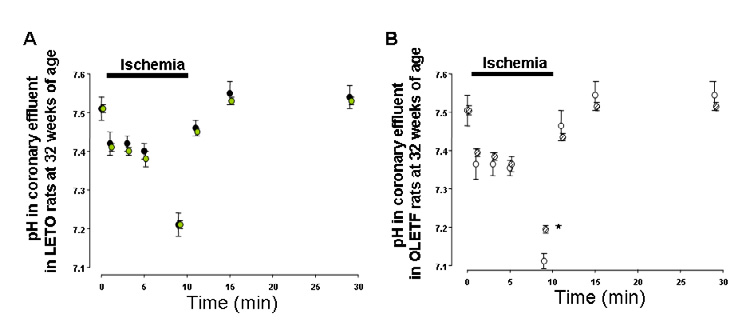
Changes in pH of coronary effluent in troglitazone-treated and non-treated LETO rats (A), and in troglitazone-treated and non-treated OLETF rats (B) at 32 weeks of age. Closed circles show non-treated LETO rats, gray circles show troglitazone-treated LETO rats, open circles show non-treated OLETF rats, bias circles show troglitazone-treated OLETF rats. * p < 0.05 vs. OLETF rats.

In all groups, pH of the coronary effluent decreased during ischemia and then recovered after ischemia. There were no significant differences in pH values of coronary effluent samples throughout the ischemia-reperfusion protocol between 16-O and 16-L (Fig. 2A), however, pH in 32-O was significantly lower than in 32-L at 9 minutes during ischemia (Fig. 2B). The pH value in 32-LT did not show a significant difference compared with that in 32-L throughout the ischemia-reperfusion protocol (Fig. 3A), though the decrease in pH was significantly suppressed in 32-OT as compared with 32-O at 5 and 9 minutes during ischemia (Fig. 5B).

### Changes in proton production in coronary effluent

**Figure 4:**
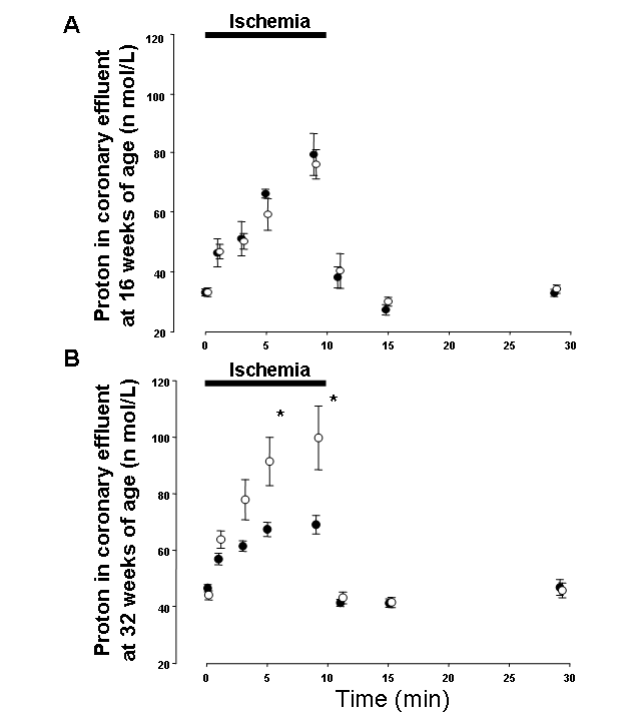
Changes in proton content in coronary effluent from LETO and OLETF rats at 16 weeks of age (A) and 32 weeks of age (B). Closed circles show LETO rats, open circles show OLETF rats. * p < 0.05 vs. LETO rats.

**Figure 5:**
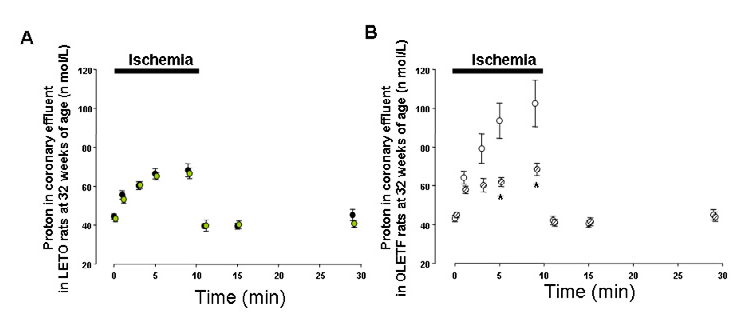
Changes in proton content in coronary effluent from troglitazone-treated and non-treated LETO rats (A), and from troglitazone-treated and non-treated OLETF rats (B) at 32 weeks of age. Closed circles show non-treated LETO rats, gray circles show troglitazone-treated LETO rats, open circles show non-treated OLETF rats, bias circles show troglitazone-treated OLETF rats. * p < 0.05 vs. OLETF rats.

Proton efflux increased during ischemia and recovered after reperfusion in all groups. No significant difference was observed between the proton efflux levels in 16-O and 16-L (Fig. 4A), whereas that in 32-O was significantly higher than that in 32-L at 5 and 9 minutes during ischemia (Fig. 4B). No significant difference was observed between 32-LT and in 32-L throughout the perfusion protocol (Fig. 5A), while the increase in proton efflux was significantly suppressed in 32-OT as compared with 32-O at 5 and 9 minutes during ischemia (Fig. 5B).

## Discussion

In the present study, a prolonged duration of reperfusion arrhythmia was observed in hearts from OLETF rats at 32 weeks of age, in which obesity and hyperglycemia were confirmed by OGTT results. Further, pH levels were lower and proton production was significantly higher in the coronary effluent of those rats as compared with age matched LETO rats. In addition, the duration of reperfusion arrhythmia was significantly shortened, while the decrease in pH and increase in proton production were significantly suppressed in coronary effluent samples following treatment with troglitazone in the hearts of those OLETF rats.

In the present study, the plasma glucose level at 60 min during OGTT was greater than 300 mg/dl in OLETF rat at 32 weeks of age, and the HbA1c level was also significantly higher as compared to the age matched LETO rats. According to our results as well as those of other studies [8,11], OLETF rats at 32 weeks of age in the present study were at the impaired glucose tolerance (IGT) stage. Demonstrating IGT and obesity, these characteristics resembled human type 2 DM at an early stage. The plasma glucose levels after fasting and in the OGTT results, as well as HbA1c and serum insulin levels, were improved in OLETF rats at 32 weeks of age by treatment with troglitazone as in other reports [12].

There have been other studies on cardiac function in diabetic OLETF rats. Mizushige et al. [3] reported the occurrence of a left ventricular diastolic dysfunction in the prediabetic stage of OLETF rat using doppler echocardiography. Abe et al. [13] also reported occurrence of left ventricular diastolic dysfunction in diabetic OLETF rats at 62–66 weeks of age in experiments with isolated hearts, though that dysfunction was not observed at 40–46 weeks of age. Impaired cardiac contraction during working heart perfusion in OLETF rats at 12 months of age [4] and an impairement in vivo cardiac contraction in OLETF rats at 36 [14] and 62 [15] weeks of age have also been reported. In the present study, the LV functional parameters before ischemia did not differ between the LETO and OLETF rats at 32 weeks of age. We considered that the discrepancies between these reports may have been caused by the different experimental methods and stage of diabetes in the animals. Meanwhile, one of the fatty obese rodents, JCR: LA-cp rats have been reported to have a normal basal myocardial function, while those animals also exhibited an increased sensitivity to ischemic myocardial injury that developed with advancing age [5]. The present results for OLETF rat hearts are similar to those findings.

The pH values in coronary effluent did not drop dramatically after inducing ischemia, in contrast to myocardial intracellular pH levels measured in other studies of no-flow ischemia [6,16]. In the present study, low-flow ischemia was induced, which might be the reason for this discrepancy. However, pH values decreased significantly after 10 min of ischemia as compared with those before ischemia in all groups (Figure 2 and 3). Additionally, those decreases in pH values after 10 min of ischemia (ΔpH = 0.3–0.4) were similar to the results of other reports with low-flow ischemia [16]. Thus, the most important finding regarding cardiac metabolism in the present study was exacerbated acidosis during ischemia in the hearts of OLETF rats after the incidence of IGT. As sources of acidosis during ischemia in hearts, anaerobic glycolysis, CO_2 _production from respiration in mitochondria, impairment in the oxygenation of NADH_2_, and the effects of proton production cycle are well-known. We previously reported higher levels of lactate production during ischemia in the hearts of OLETF rats at 32 weeks of age [17]. Thus, the findings in the present study may mainly correlate with an acceleration of anaerobic glycolysis during ischemia in the myocardium of OLETF rats at that age. Anaerobic glycolysis is an important source of ATP production, however, it causes increases in the accumulation of acidotic metabolites and decreases in intracellular pH, and is also correlates with the severety of myocardial damage. In addition, local acidosis leads to alterations in electrical currents and electrophysiological changes in cell membranes [18]. While, severe acidosis and elevated proton production increase intracellular Na^+ ^via Na^+^-H^+ ^exchanger during ischemia in the myocardium. Increased intracellular Na^+ ^causes intracellular Ca^2+ ^overload after reperfusion, and reperfusion arrhythmia occurs [19]. Regarding these previous reports, exacerbation of acidosis and increased proton production can be correlated with prolonged duration of reperfusion induced VF in the hearts of OLETF rats at 32 weeks of age. Additionally, IGT improved, and exacerbation of acidosis and an increase in proton production in the coronary effluent were suppressed during ischemia at that age by treatment with troglitazone. Moreover, the duration of reperfusion induced VF was shortened simultaneously. These results confirmed the correlation between impaired glucose metabolism and increased sensitivity to ischemia in the myocardium of OLETF rats after the incidence of IGT. In ischemia-reperfusion experiments using spontaneous type 2 diabetic rodent hearts, impaired recovery of cardiac function after reperfusion has been reported [4]. In a heart perfusion study conducted by Maddaford et al. [5], ATP and glycogen contents in the insulin-resistant rat myocardiums decreased significantly after ischemia-reperfusion, indicating acceleration of the consumption and drying of stored energy. Sidell et al [6] reported a greater loss of ATP and lactate production in the Zucker Fatty rat hearts during ischemia, thus correlating to a lower recovery in the contractile function during reperfusion. In the only known report of reperfusion arrhythmia in spontaneous type 2 diabetic animal hearts, which utilized *db/db *mice, an increase in the incidence and prolonged duration of reperfusion arrhythmia were observed [7]. In that study, a greater increase in intracellular Na^+ ^was indicated as the main cause of those conditions. As for ion homeostasis, altered intracellular Ca^2+ ^handling [20] and K_ATP _currents [21] in spontaneous diabetic mouse myocytes have been reported. These alterations also have possibility to increase the incidence or prolong the duration of reperfusion arrhythmia. Thus, some metabolic mechanisms or ion homeostasis apart from intracellular acidosis and proton efflux may correlate with increased sensitivity to ischemia in hearts of OLETF rats at 32 weeks of age.

We also observed a shortening of the duration of reperfusion arrhythmia VF following treatment with troglitazone in the hearts of non-diabetic LETO rats at 32 weeks of age. There was not a significant difference in pH level and proton efflux between the treated and non-treated groups. Then, some mechanisms apart from improvement of glucose metabolism might shorten the duration of the reperfusion induced VF in the hearts of OLETF rats at 32 weeks of age treated with troglitazone. In fact, an improvement in the recovery of cardiac function after ischemia has been reported in non-diabetic pig hearts treated with troglitazone for a long time [22]. However, after treatment with troglitazone, the incidence of VF did not improve in the hearts of LETO rats at 32 weeks of age, whereas that in the hearts of age matched OLETF rats tended to be improved (p = 0.10). These results demonstrate that suppression of alteration in glucose metabolism according to the incidence of IGT in myocardium should be the main mechanism improving the exacerbation of reperfusion injury by treatment with troglitazone in OLETF rat. Recent clinical trials have revealed that thiazolinediones provide a better prognosis in diabetic patients with coronary heart disease [23]. However, the effects of thiazolidinediones, including troglitazone, on the heart have not been well clarified and additional investigations are required.

## Conclusion

Sensitivity to ischemia increased after the incidence of impaired glucose tolerance in the hearts of OLETF rats, which improved by the treatment with troglitazone. We concluded that exacerbation of acidosis through altered glucose metabolism during ischemia is one of the causes of this phenomenon in diabetic OLETF rats.

## Abbreviations

DM, diabetes mellitus; OGTT, oral glucose tolerance test; VF, ventricular fibrillation.

## References

[B1] Haffner SM, Lehto S, Ronnemaa T, Pyorala K, Laakso MM (1998). Mortality from coronary heart disease in subjects with type 2 diabetes and in nondiabetic subjects with and without prior myocardial infarction. N Engl J Med.

[B2] Devereux RB, Roma MJ, Paranicas M, O'Grady MJ, Lee ET, Welty TK, Fabsitz RR, Robbins D, Rhoades ER, Howard BV (2000). Impact of diabetes on cardiac structure and function. The Strong Heart Study. Circulation.

[B3] Mizushige K, Yao L, Noma T, Kiyomoto H, Yu Y, Hosomi N, Ohmori K, Matsuo H (2000). Alteration in left ventricular diastolic fillingand accumulation of myocardial collagen at insulin-resistantprediabetic stage of a type II diabetic rat model. Circulation.

[B4] Chen H, Higashino H, Kamenov ZA, Azuma M, Lee WH, Yang XQ, Zhou DJ, Yuan WJ (2003). Preserved post ischemic heart function in sucrose-fed type 2 diabetic OLETF rats. Life Sciences.

[B5] Maddaford TG, Russell JC, Pierce GN (1997). Postischemic cardiac performance in the insulin-resistant JCR: LA-cp rat. Am J Physiol.

[B6] Sidell RJ, Cole MA, Draper NJ, Desrois M, Buckingham RE, Clarke K (2002). Thiazolidinedione treatment normalizes insulin resistance andischemic injury in the Zucker Fatty rat heart. Diabetes.

[B7] Anzawa R, Bernard M, Tamareille S, Baetz D, Confort-Gouny S, Gascard JP, Cozzone P, Feuvray D (2006). Intracellular sodium increase and susceptibility to ischaemia in hearts from type 2 diabetic *db/db *mice. Diabetologia.

[B8] Kawano K, Hirashima T, Mori S, Saitoh Y, Kurosumi M, Natori T (1992). Spontaneous long-term hyperglycemic rat with diabetic complications. Otsuka Long-Evans Tokushima Fatty (OLETF) strain. Diabetes.

[B9] Nolan JJ, Ludvik B, Beerdsen P, Joyce M, Olfsky J (1994). Improvement in glucose tolerance and insulin resistance in obese subjects treated with troglitazone. N Eng J Med.

[B10] Walker MJA, Curtis MJ, Hearse DJ, Campbell RWF, Janse MJ, Yellon DM, Cobbe SM, Coker SJ, Harness JB, Harron DWG, Higgins AJ, Julian DG, Lab MJ, Manning AS, Northover BJ, Parratt JR, Riemersma RA, Riva E, Russell DC, Sheridan DJ, Winslow E, Woodward B (1988). The Lambeth Conventions: guidelines for the study of arrhythmias in ischemia, infarction, and reperfusion. Cardiovasc Res.

[B11] Yagi K, Kim S, Wanibuchi H, Yamashita T, Yamamura Y, Iwao H (1997). Characteristics of diabetes, blood pressure and cardiac and renal complications in Otsuka Long Evans Tokushima Fatty rats. Hypertension.

[B12] Jia DM, Tabaru A, Nakamura H, Fukumitsu KI, Akiyama T, Otsuki M (2000). Troglitazone prevents and reverses dyslipidemia, insulin secretory defects, and histologic abnormalities in a rat model of naturally occurring obese diabetes. Metabolism.

[B13] Abe T, Ohga Y, Tabayashi N, Kobayashi S, Sakata S, Misawa H, Tsuji T, Kohzuki H, Suga H, Taniguchi S, Takaki M (2002). Leftventricular diastolic dysfunction in type 2 diabetes mellitus modelrats. Am J Phisiol.

[B14] Hayashi T, Sohmiya K, Ukimura A, Endoh S, Mori T, Shimomura H, Okabe M, Terasaki F, Kitaura Y (2003). Angiotensin II receptor blockade prevents microangiopathy and preserves diastolic function in the diabetic rat heart. Heart.

[B15] Saito F, Kawaguchi M, Izumida J, Asakura T, Maehara K, Maruyama Y (2003). Alteration in haemodynamics and pathological changes inthe cardiovascular system during the development of Type 2 diabetes mellitus in OLETF rats. Diabetologia.

[B16] El Banani H, Bernard M, Baets D, Cabanes E, Cozzone P, Lucien A, Feuvray D (2000). Changes in intracellular sodium and pH during ischaemia-reperfusion are attenuated by trimetazine. Comparison between low- and zero-flow ischaemia. Cardiovasc Res.

[B17] Anzawa R, Seki S, Horikoshi K, Onodera T, Taniguchi M, Mochizuki S (1999). Ischaemic tolerance is attenuated in the spontaneously diabetic Otsuka Long-Evans Tokushima Fatty rats. Eur Heart J.

[B18] Cascio WE, Johnson TA, Gettes LS (1995). Electrophysiologic changes in ischemic ventricular myocardium: I. Influence of ionic, metabolic and energic changes. J Cardiovasc Electrophysiol.

[B19] Lu HR, Yang P, Remeysen P, Saels A, Dai DZ, De Clerck F (1999). Ischemia/reperfusion induced arrhythmias in anaesthetized rats: a role of Na^+ ^and Ca^2+ ^influx. Eur J Pharmacol.

[B20] Pereira L, Matthes J, Schuster I, Valdivia HH, Herzig S, Richard S, Gomez AM (2006). Mechanisms of [Ca^2+^]_i _transient decrease in cardiomyopathy of *db/db *type 2 diabetic mice. Diabetes.

[B21] Shimoni Y, Chuang M, Abel ED, Severson DL (2004). Gender-dependent attenuation of cardiac potassium currents in type 2 diabetic *db/db *mice. J Physiol.

[B22] Zhu P, Lu L, Xu Y, Schwartz GG (2000). Troglitazone improves recovery of left ventricular function after regional ischemia in pigs. Circulation.

[B23] Dormandy JA, Charbonnel B, Eckland DJ, Erdmann E, Massi-Benedetti M, Moules IK, Skene AM, Tan MH, Lefèbvre PJ, Murray GD, Standl E, Wilcox RG, Wilhelmsen L, Betteridge J, Birkeland K, Golay A, Heine RJ, Korányi L, Laakso M, Mokán M, Norkus A, Pirags V, Podar T, Scheen A, Scherbaum W, Schernthaner G, Schmitz O, Skrha J, Smith U, Taton J, PROactive investigators (2005). Secondary prevention of macrovascular events in patients with type 2 diabetes in the PRO active Study (PROspective pioglitAzone Clinical Trial In macroVascular Events): a randomized controlled trial. Lancet.

